# Comparing variant calling algorithms for target-exon sequencing in a large sample

**DOI:** 10.1186/s12859-015-0489-0

**Published:** 2015-03-07

**Authors:** Yancy Lo, Hyun M Kang, Matthew R Nelson, Mohammad I Othman, Stephanie L Chissoe, Margaret G Ehm, Gonçalo R Abecasis, Sebastian Zöllner

**Affiliations:** 1 Department of Biostatistics, University of Michigan, 1415 Washington Heights, Ann Arbor, MI 48109 USA; 2 GlaxoSmithKline, Quantitative Sciences, Research Triangle Park, NC USA; 3 Department of Ophthalmology, University of Michigan, Ann Arbor, MI USA; 4 Department of Psychiatry, University of Michigan, Ann Arbor, MI USA

**Keywords:** Next-generation sequencing, Targeted sequencing, Variant calling

## Abstract

**Background:**

Sequencing studies of exonic regions aim to identify rare variants contributing to complex traits. With high coverage and large sample size, these studies tend to apply simple variant calling algorithms. However, coverage is often heterogeneous; sites with insufficient coverage may benefit from sophisticated calling algorithms used in low-coverage sequencing studies. We evaluate the potential benefits of different calling strategies by performing a comparative analysis of variant calling methods on exonic data from 202 genes sequenced at 24x in 7,842 individuals. We call variants using individual-based, population-based and linkage disequilibrium (LD)-aware methods with stringent quality control. We measure genotype accuracy by the concordance with on-target GWAS genotypes and between 80 pairs of sequencing replicates. We validate selected singleton variants using capillary sequencing.

**Results:**

Using these calling methods, we detected over 27,500 variants at the targeted exons; >57% were singletons. The singletons identified by individual-based analyses were of the highest quality. However, individual-based analyses generated more missing genotypes (4.72%) than population-based (0.47%) and LD-aware (0.17%) analyses. Moreover, individual-based genotypes were the least concordant with array-based genotypes and replicates. Population-based genotypes were less concordant than genotypes from LD-aware analyses with extended haplotypes. We reanalyzed the same dataset with a second set of callers and showed again that the individual-based caller identified more high-quality singletons than the population-based caller. We also replicated this result in a second dataset of 57 genes sequenced at 127.5x in 3,124 individuals.

**Conclusions:**

We recommend population-based analyses for high quality variant calls with few missing genotypes. With extended haplotypes, LD-aware methods generate the most accurate and complete genotypes. In addition, individual-based analyses should complement the above methods to obtain the most singleton variants.

**Electronic supplementary material:**

The online version of this article (doi:10.1186/s12859-015-0489-0) contains supplementary material, which is available to authorized users.

## Background

With rapid advances in sequencing technology, large-scale sequencing studies enable discovery of rare polymorphisms. Exome and targeted sequencing studies are especially popular in the studies of complex traits. These designs focus on small genome regions likely to be enriched for functional variants [1-3], achieving higher coverage of an important subset of the genome and facilitating larger sample sizes [4,5]. While variant calling typically improves with increasing read coverage [6], exome and targeted experiments tend to generate uneven coverage. For studies averaging 40x to 120x, empirical coverage per targeted position per sample can range from less than 5x to over 150x [7-10]. At high coverage, genotypes can be called with high precision using basic calling strategies [3]. However, at regions with local low coverage, calling genotypes accurately is challenging, leading to more errors and missing data [11]. In studies with low mean coverage, advanced variant calling algorithms compensate by combining read information with linkage disequilibrium (LD) information across large samples [12,13]. However, it is unclear if such algorithms substantially improve genotypes in datasets with heterogeneous coverage. To address this question, we evaluated the performance of advanced variant calling algorithms in targeted sequencing experiments. Our goal was to provide specific guidelines for applying variant calling algorithms to these studies.

Variant calling algorithms fall into three major categories depending on how information from shotgun sequencing data is aggregated across individuals and genomic positions [14]. The first category involves individual-based single marker callers (IBC), which assign genotypes based on aligned reads from a single individual at a single position [15-19]. These callers are typically applied to high-depth exome sequencing data [7,8]. The second category of algorithms is population-based single marker callers (PBC), where reads per position from all samples jointly determine polymorphism and allele frequencies. Based on estimated allele frequencies, these methods then call genotypes using per individual read data [11,20]. PBC is typically used in low-pass sequencing studies [12,21,22]. The third category of calling algorithms utilizes linkage disequilibrium (LD) information across several hundred kilobases flanking each variant base identified by an IBC or PBC [12,23,24]. Similar to widely used imputation algorithms [25], these LD-aware calling methods (LDC) phase existing variant calls into haplotypes, then update genotypes according to the joint evidence across similar haplotypes. LDC, though computationally demanding, have been used in combination with PBC to successfully interpret low-coverage, genome-wide data such as that in the 1000 Genomes Project [21,22].

To compare the performance of the three types of algorithms in large-scale sequencing datasets with high coverage, we analyzed 7,842 European individuals, each sequenced at 202 targeted genes [26]. The average per targeted site per individual coverage was 24x, but with a wide range from 0 to >75x (Additional file 1: Figure S1a). Genotype data from previous genome-wide association studies (GWAS) provided long haplotypes for LD-aware genotype calling. We generated four sets of variant calls from this dataset, using (1) IBC, (2) PBC, (3) LDC based on only the sequencing data and (4) LDC after combining the sequencing data with flanking GWAS data. We focused on a fixed number of variants per call set after ranking the variants by quality control metrics, and assessed the quality of each filtered call set by transition to transversion ratio and the percentage of called variants confirmed in SNP databases. Moreover, we evaluated genotype accuracy by collating 80 pairs of experimental replicates and by comparing sequencing calls with on-target genotypes from previous GWASs. We further validated a subset of caller-specific singletons at the heterozygous individuals with an independent capillary sequencing experiment. Finally, to ensure applicability of our comparison findings to other studies, we investigated our dataset using alternative approaches of IBC and PBC. We also generated IBC variant calls from an additional dataset with average coverage of 127.5x, sequenced at 57 genes from 3,142 individuals [10], and compared these calls with an existing PBC call set.

We found that at a fixed number of variant sites, IBC identified a larger proportion of extremely rare variants of high quality, particularly singletons, while capturing most of the common polymorphic sites that were identified by the other callers. We replicated the result in the additional high-coverage dataset and by using different variant caller implementations. However, IBC genotypes at common variants were of the lowest quality by all measures. They were the least concordant with GWAS genotypes and within sequencing replicate pairs. Moreover, the IBC call set contained 4.72% missing genotypes, due to low coverage or low quality calls. In the PBC set, the percentage of missing genotypes dropped to 0.47% by using a population allele frequency prior. PBC also showed improved heterozygous concordance with on-target GWAS genotypes as well as between replicates. Without flanking markers, LDC achieved similar genotype accuracy with PBC, while further reducing the missing genotypes to 0.17%. With extended haplotypes from flanking GWAS markers, LDC achieved the same level of missing genotypes (0.17%) and the highest genotype concordance among all callers.

## Methods

### Data description

To understand the strengths and limitations of individual-based, population-based and LD-aware variant calling methods, we analyzed sequence read data from 7,842 unrelated European individuals. The next-generation sequencing data was part of a large-scale targeted sequencing experiment generated for the purpose of identifying variants associated with 12 common diseases and cardiovascular and metabolic phenotypes, previously described in Nelson *et al*. [26]. This experiment targeted 2,218 exons of 202 genes of potential drug interest, covering 864kb (~1%) of the coding genome. Each exon was captured to include the coding sequence plus UTR and 50 bp flanking sequence on each end. Each sample had on average 0.6 million 100 bp paired-end Illumina reads, with overall average depth of 24x, but depth averaged per individual per targeted site ranged from 0x to over 75× (Additional file 1: Figure S1a). In particular, six genes had low mean coverage (<10×) across all exons; the mean coverage across gene regions and across individuals spanned a range of 7× to 35× (Additional file 1: Figure S1b).

Among the 7,842 individuals considered, 80 were independently sequenced twice. All 7,842 individuals had been previously typed on one of Illumina (300k, 550k, 610k) or Affymetrix (500k, 6.0) chips for genome-wide association studies (GWASs). Prior to variant calling, we aligned reads using BWA 0.5.9 (http://bio-bwa.sourceforge.net) [27] with human genome build 36 as reference. We removed duplicate reads using Picard (http://picard.sourceforge.net/). We recalibrated base quality scores using Genome Analysis Toolkit (1.0.5974) from the Broad Institute [18]. We combined the GWAS genotype data from various chips using PLINK [28] (Additional file 1).

### Variant calling

We used likelihood-based models for genotype and SNP calling, as outlined in Li *et al.* [12]. For each of the 10 possible genotypes (AA, AC, AT, AG, CC, CT, CG, TT, TG, GG) at each locus, the model computes genotype likelihood *Pr(reads|genotype)*. These likelihoods are calculated per genomic position with aligned reads. Base quality scores of the reads are refined using the base alignment quality (BAQ) adjustment to account for base calling error rates and mapping uncertainty [29]. Using Bayes' rule, these likelihoods are combined with a model-specific prior on the genotype *π(genotype)* to generate posterior probabilities *Pr(genotype|reads)*. We considered 3 categories of calling algorithms that reflect how information is aggregated across individuals and positions.

### Individual-based single marker caller (IBC)

IBC applies an individual-based prior which assumes each allele has a probability θ = 0.001 of being different from the reference. For variant sites, we assigned uniform prior probabilities for transitions and transversions to avoid bias in the evaluation of genotype quality based on transition to transversion ratio (Ts/Tv). By computing the genotype likelihoods using aligned reads per individual, the model assigns the most likely genotype when the posterior probability reaches a threshold of 99%; genotypes with lower posterior probability are marked as missing. We used glfSingle (http://genome.sph.umich.edu/wiki/GlfSingle) to call genotypes. By calling also the reference homozygous genotypes, we obtained the union set of all variant sites and genotypes across all individuals.

### Population-based single marker caller (PBC)

PBC uses a two-step procedure to call variants [12]. First, upon observing at least one read carrying a non-reference allele, the model applies a population genetic prior that estimates the probability of the site being polymorphic as a function of sample size, with per base pair heterozygosity of θ = 0.001 under the stationary neutral model [30]. As with IBC, the model assumes a prior with uniform Ts/Tv. Second, per polymorphic site, PBC estimates the population allele frequency *f* using aligned reads from all individuals, assuming a biallelic site in Hardy-Weinberg equilibrium. These allele frequency priors combine with the likelihoods calculated per individual to generate posterior genotype probabilities. We used the PBC implemented as glfMultiples (http://genome.sph.umich.edu/wiki/GlfMultiples), which also generated variant calls for NHLBI GO Exome Sequencing Project (ESP) and contributed to 1000 Genomes Project analyses [21,22,31].

In this study, we used a posterior probability threshold of 99% for the most likely genotype, which was the same threshold as for the ESP [31]. To maintain independence between experimental replicates, we generated two call sets, each including 7,762 unique samples plus 80 samples, one from each sequence replicate pair.

### LD-aware caller (LDC)

Starting from a set of variant calls, LDC updates the genotype of each individual at each marker using a Hidden Markov Model derived from the haplotype-based model used in the imputation software MACH [32]. The LDC algorithm starts with randomly phased haplotypes for each individual. Per iteration, the algorithm compares one sequenced sample with a randomly picked subset of haplotypes. It updates each genotype or imputes missing genotypes, based on the similarity of the sample haplotype to the reference haplotypes. In addition to identifying the most likely genotype, LDC calculates the expected number of reference alleles carried by each individual (dosage). Per variant site, LDC also estimates the correlation coefficient *R* ^*2*^ between true allele counts and estimated allele counts, as a measure of imputation quality. This caller, previously used in low-pass sequencing studies [12,22], has been implemented as ThunderVCF (http://genome.sph.umich.edu/wiki/ThunderVCF).

We used LDC to refine each of the two PBC call sets described above. We applied the standard setting of 30 iterations and 200 reference haplotypes per iteration. We considered two scenarios with different haplotype information: First, we applied LDC on short haplotypes, which consisted only of the PBC variant calls at the sequences captured in the sequencing experiment. Second, we created long haplotypes by combining PBC variant calls with GWAS-genotypes from flanking markers within 500 kb from both ends of each target gene. In both scenarios, we masked GWAS genotypes within the target regions and used these markers as measures of genotype quality.

### Variant quality control

To remove potentially false variant calls caused by technical artifacts, we followed the filtering and support vector machine (SVM) approach used in the ESP [31] and Zhan *et al*. [10]. Initial filtering included quality metrics based on read alignments, nearby indels and excess heterozygosity (Additional file 1). For LD-aware calls, we imposed an additional *R* ^*2*^ quality control criterion, which filters sites with ${\widehat{R}}^2<0.7$.

SVM generates a summary score for each site based on the initial quality metrics, classifying good and bad calls with respect to training call sets (Additional file 1). We ranked these scores and selected the 27,500 top-ranked variants per call set for comparison. We set the cutoff to compare only variants with positive SVM scores.

After selecting 27,500 top-ranked variants per call set from SVM classification, we filtered individual genotypes of each variant to discard those with more than 1% estimated error. From IBC genotypes, we removed and marked as missing the genotypes with PHRED quality score less than 20 or with genotype depth less than 7x. As the quality of PBC genotypes is less affected by individual genotype depth, we only filtered with PHRED quality < 20. Analogously, we filtered LD-aware genotypes with a posterior probability ratio < 99:1 between the genotypes with the highest and the second highest posterior probability.

### Comparing call sets

We compared 4 sets of 27,500 variants, generated using IBC, PBC, LDC without flanking haplotypes and LDC with flanking haplotypes. First, we evaluated the overall quality of each call set by calculating transition to transversion ratios (Ts/Tv), stratified by variant type as annotated by ANNOVAR (hg19, gencodeV7, http://www.openbioinformatics.org/annovar/) [33] and by minor allele count. Second, we compared our call sets to the Single Nucleotide Polymorphism database (dbSNP, release 135, http://www.ncbi.nlm.nih.gov/SNP/), a recent public archive of confirmed variants.

We then characterized IBC-specific variants and PBC-specific variants by their Ts/Tv and read coverage. Most of the IBC- and PBC-specific variants were singletons. We performed an independent capillary sequencing experiment on 32 IBC-specific and 41 PBC-specific singleton variants, sampled from individuals from the CoLaus study [26] carrying the singleton heterozygous genotypes (Additional file 1). Error rates from this validation provided estimates of false discovery rates of caller-specific singletons. Finally, we extended the validation to 51 caller-specific singletons with SVM scores below the cutoff, to assess the quality of discarded sites from each set.

We assessed genotype quality of each call set by four summary statistics: (1) The percentage of missing genotypes from no calls and filtered genotypes (2) The pairwise heterozygote mismatch rates (*h*_*e*_) between our genotype calls from sequencing and the genotypes from GWAS chips at the on-target markers. *h*_*e*_ is defined as the number of genotypes called as heterozygous in one set but homozygous in the other, divided by the total number of heterozygous genotypes in both sets. (3) *h*_*e*_ for the 80 sequence replicate pairs, at variant sites where at least one individual per pair is heterozygous. (4) The shared variants between each pair of call sets and calculated the *h*_*e*_ between every pair of callers.

To investigate the effect of sample size on the difference in performance between IBC and PBC, we performed down-sampling analyses on our original dataset, evaluating the ability of the PBC caller to identify variants called as singletons by IBC. For simplicity, we focused on variants that were called as singletons in the full dataset of 7,842 individuals (IBC singletons). We generated random samples of 50, 100, 500, 1,000, 2,500 and 5,000 individuals from the original dataset by sequentially adding individuals and used PBC to call variants in each of the samples. For each down-sampled dataset, we calculated the proportion of IBC singletons identified by PBC and recorded the genotype quality of these PBC singletons. We repeated the full random sampling experiment 10 times and averaged the results.

To assess if our results were driven by the specific choice of calling algorithms, we applied the individual- and population-based settings of GATK UnifiedGenotyper (version 3.1.1-g07a4bf8) [11] to our original dataset. The UnifiedGenotyper follows the same genotype likelihood framework described above for variant calling. In particular, it uses the same model for individual- and population-based calling, where it estimates simultaneously the population allele frequency and most likely genotypes. To generate individual-based calls, population size is set to 1. We generated individual- and population-based variants for our targeted exon data with 7,842 samples. We compared the two resulting call sets, focusing on the singletons specific to each analysis.

To replicate our results in a second dataset with higher sequencing coverage, we considered an additional dataset obtained from the AMD Consortium, which sequenced 3,142 individuals at 57 genes from 10 age-related macular degeneration loci [10]. The average coverage was 127.5x, but 10% of the genes suffered from low average coverage of around 10x (Additional file 1: Figure S2). We generated IBC variant calls and compared them with existing PBC variant calls of this dataset, obtained from the project investigators. We evaluated the IBC-specific singletons, particularly those at sites with local low coverage, and contrasted them with singletons identified by IBC and PBC.

## Results

### Summary of variant call sets

In the complete call sets of 7,842 individuals, the individual-based single marker caller (IBC) generated 31,970 variants while the population-based single marker caller (PBC) generated 29,147 variants. The LD-aware caller (LDC) modified genotypes from PBC, hence it generated the same number of variants. We filtered each call set separately and ranked the variants using a support vector machine (SVM). We observed 30,297 IBC, 27,690 PBC variants and 27,535 LDC variants with positive SVM scores. To compare call sets for a fixed call rate, we focused on the top 27,500 variant sites from each set. In the IBC set, 59.4% of the calls were singletons (MAF = 0.06%), while 57.7% of the PBC and LDC calls were singletons (Table 1). Over 81% of variants in each call set had minor allele counts ≤ 5. Most of these rare variants were novel; only 26-27% of variants from each call set were recorded in the dbSNP database (Table 1).

**Table 1:** **Summary statistics of 27,500 top-ranked SNPs per call set and quality assessed by transition-to-transversion ratio (Ts/Tv) and missing genotypes**

		**All SNPs**				**Singletons**		**%Missing genotypes**
**Call set**	**#SNPs**	**% dbSNP**	**Known Ts/Tv**	**Novel Ts/Tv**	**Overall Ts/Tv**	**#SNPs**	**Ts/Tv**	
IBC	27500	25.72%	3.02	2.54	2.71	16325 (59.36%)	2.57	4.71
PBC	27500	26.87%	3.02	2.45	2.59	15877 (57.73%)	2.44	0.47
LDC	27500	26.85%	3.01	2.45	2.59	15857 (57.66%)	2.44	0.17
LDC + F	27500	26.81%	3.00	2.45	2.58	15869 (57.71%)	2.44	0.17

*Abbreviations:* IBC = individual-based single marker caller, PBC = population-based single marker caller, LDC = LD-aware caller without flanking haplotypes, LDC + F = LD-aware caller with flanking haplotypes. Expanded table showing quality of call sets broken down by variant class is included in Additional file 1: Table S1.

Combining our four filtered call sets each of 27,500 SNPs, our analyses generated a total of 29,652 autosomal SNPs. We identified 1,035 variants not previously found in the Nelson *et al*. analyses of the same dataset [26]. Among these, 509 (48.16%) were IBC-specific, while 445 (42.10%) were in all call sets (Additional file 2: Database S1).

The IBC call set had the highest percentage of missing genotypes (4.72%), while the PBC call set had a substantially lower percentage (0.47%) (Table 1). The LDC call set had the lowest percentage of missing genotypes (0.17%). Typically LDC genotypes have no missing data; in our analysis, missing genotypes in LDC were a result of filtering genotypes with more than 1% uncertainty.

### Overall quality of variant call sets

We assessed the quality of the variants included in the four call sets by calculating the transition-to-transversion ratio (Ts/Tv). A Ts/Tv > 2 is expected for intergenic sites; Ts/Tv is typically much higher in coding regions due to purifying selection [31]. In our data, Ts/Tv of the unfiltered IBC call set was 2.27, and Ts/Tv of the unfiltered PBC and LDC call sets were both 2.46. Ts/Tv of all call sets increased after SVM classification at the 27,500 variant cutoff (Table 1), indicating reasonable quality control. We then focused on the quality of these SVM top-ranked call sets. As Table 1 shows, the IBC call set attained the highest Ts/Tv of 2.71, while PBC and LDC without flanking haplotypes had a Ts/Tv of 2.59. LDC with flanking haplotypes had a Ts/Tv of 2.58.

Comparing Ts/Tv between known variants and novel variants, we observed that known variants (in dbSNP) generally had higher Ts/Tv than novel variants (Table 1). Singletons had slightly lower Ts/Tv compared to the corresponding overall call set, as singletons represent recent mutations that are less affected by purifying selection [34]. Analogously, known variants had a higher Ts/Tv because such variants are typically older and have been subjected to purifying selection for longer.

At exonic variants, all call sets attained Ts/Tv greater than 3, with nonsynonymous variants having lower Ts/Tv than synonymous variants (Additional file 1: Table S1). The coding variants had higher Ts/Tv than non-coding variants in all call sets, because coding sequences contains higher proportion of CpG sites enriched for transitions compared to non-coding regions, and because transitions are enriched at degenerate sites within coding regions. Intergenic and flanking variants had Ts/Tv around 2 in all call sets, consistent with expectations (Additional file 1: Table S1).

### Evaluating singleton variants

Most caller-specific variants were singletons. We found 4,203 caller-specific variants out of 29,652 in the union call set. Of these, 1,850 (44.02%) were IBC-specific, 1,787 (96.59%) being singletons with Ts/Tv 1.97. On the other hand, 1,731 (41.18%) variants were shared between PBC and LDC sets, but not found by IBC. We considered sites in this category as PBC-specific since LDC did not introduce new sites, but only modified genotypes at sites called by PBC. Of these PBC-specific sites, 1,260 (72.79%) were singletons with Ts/Tv 1.08.

**Figure 1:**
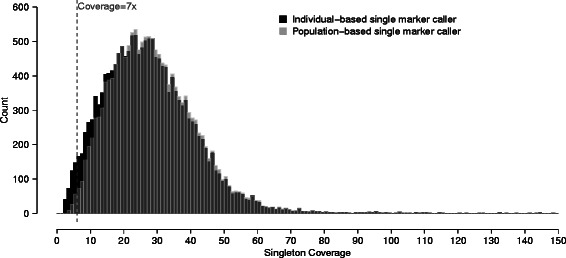
**Distribution of coverage at the individual carrying the singleton alternative allele.** We compare the distribution of coverage at called singleton variants between individual-based caller (black) and population-based caller (light gray). The overlap of the two distributions is in dark gray. Here we show all singleton variants after SNP filtering and genotype filtering on quality < 20. We keep individual-based single marker calls at low genotype coverage for this comparison, with the vertical dash line indicating genotype coverage filter at 7x.

IBC identified more singletons at low coverage than PBC, even after an additional filtering of all genotypes with less than 7x coverage (Figure 1). Independent capillary sequencing experiment validated 30 out of 30 (100%) IBC-specific singletons, and 38 out of 41 (92.68%) PBC-specific singletons (Additional file 1: Table S2). This difference in validation rates was not statistically significant (Fisher’s exact *p*-value = 0.258). Relaxing the SVM threshold to 29,000 SNPs per call set, IBC-specific and PBC-specific singletons still had comparable validation rates, at 91.30% (42/46) and 92.45% (49/53) respectively.

Notably, 99.13% of PBC-specific sites were in the IBC unfiltered (complete) call set of 31,970, including all 471 sites with minor allele count >1. On the other hand, only 177 (9.57%) IBC-specific sites were in the PBC complete call set of 29,147; the majority was undiscoverable using PBC. Therefore, we extended the validation experiment to IBC-specific singleton calls ranked below 29,000, where no singletons from PBC could be sampled from the CoLaus subset. Capillary sequencing showed that these IBC-specific singletons at the lowest ranks had a validation rate of 81.82% (18/22; Additional file 1: Table S2).

To compare the performance of singleton calling between IBC and PBC in a different dataset with higher average coverage, we repeated these analyses on a targeted sequencing dataset of 3,142 individuals sequenced at a mean coverage of 127.5x [10]. We generated an IBC call set which contained 33,615 variants with Ts/Tv 2.12, while the existing PBC call set contained 31,527 variants with Ts/Tv 2.10. Comparing these two call sets, IBC called 1,913 more singletons than PBC. These additional singletons had Ts/Tv 1.63. Interestingly, the additional singletons with high quality were located in regions with low coverage. At depth <10x and with an extra genotype quality filter of >10, IBC identified 864 additional singletons with Ts/Tv 2.18. At the same genotype depth and quality thresholds, IBC and PBC shared 911 singleton variant calls with Ts/Tv 2.13 (Additional file 1: Figure S3). When we relaxed the genotype depth threshold to < 20x, IBC identified 1,360 additional singletons with Ts/Tv 1.90, while IBC and PBC shared 2,745 singletons with Ts/Tv 2.07.

We evaluated the impact of sample size on the difference in performance between IBC and PBC by down-sampling the data to sample sizes of 1, 50, 100, 500, 1,000, 2,500 and 5,000 and calling variants in these smaller datasets using PBC. We compared the PBC singletons from each down-sampled set to high-quality IBC singletons from the original dataset of sample size 7,842. We observed that for sample sizes > 1, PBC failed to identify all IBC singletons. The proportion of IBC singletons called by PBC decreased as sample size increased. The quality score of the singletons called by PBC also decreased with sample size. At sample size = 100, PBC called 89.6% of the IBC singletons with average quality score of 73.7; at sample size = 5,000, the percentage dropped to 84.0% with average singleton quality score 69.5 (Additional file 1: Figure S4).

### Evaluating non-singleton variants

We assessed genotype quality of common variants by comparing genotypes at 378 on-target variants shared between all call sets and the GWAS data from the same individuals (Table 2a). The IBC call set had the highest discordance with GWAS genotypes, with heterozygous mismatch *h*_*e*_ = 0.82% discordant genotypes. While heterozygous mismatch rates were comparable between PBC and LDC with no flanking haplotypes, at *h*_*e*_ = 0.38% and 0.39% respectively, the rate was lower for LDC with flanking haplotypes, at 0.32% (Table 2a).

**Table 2:** **Heterozygous mismatch (a) between sequence calls and GWAS genotypes at 378 on-target GWAS markers, (b) between 80 sequence replicate pairs and (c) between pairs of algorithms**

		**Heterozygous mismatch rate**			
		**IBC**	**PBC**	**LDC**	**LDC + F**
(a)	All samples at 378 GWAS markers	0.82%	0.38%	0.39%	0.32%
(b)	80 sequence replicate pairs at all called variants	1.01%	0.34%	0.36%	0.20%
(c)	Pairwise comparison of callers				
	vs PBC	0.42%	--	--	--
	vs LDC	0.93%	0.35%	--	--
	vs LDC + F	1.01%	0.41%	0.30%	--

**Table 3:** **Heterozygous mismatch (a) between each call set and GWAS genotypes at 378 on-target markers, and (b) between additional heterozygous genotypes in more complex algorithms and the GWAS markers**

**All samples at 378 GWAS markers**			**IBC**	**PBC**	**LDC**	**LDC + F**
(a)	Number of heterozygous genotypes (hets)		276,761	293,730	298,220	298,531
	Heterozygous mismatch		0.82%	0.38%	0.39%	0.32%
(b)	Number of additional hets and heterozygous mismatch	not in IBC	–	15,727 (0.85%)	17,937 (1.23%)	18,308 (0.47%)
		not in PBC	–	–	3,113 (2.41%)	3,664 (0.71%)
		not in LDC	–	–	–	1,145 (0.87%)

Genotype concordance between sequencing replicate pairs provided a second metric of robustness of each calling algorithm (Table 2b). *h*_*e*_ at replicate pairs followed the same qualitative trend as the GWAS comparison (Table 2a), where IBC had the highest *h*_*e*_ = 1.01% at replicate pairs. The heterozygous mismatch rates were 0.34% for PBC and 0.36% for LDC without flanking haplotypes. With flanking haplotypes, *h*_*e*_ = 0.20% between experimental replicates of LDC. This mismatch rate was lower than the *h*_*e*_ with GWAS genotypes, suggesting that the error rate of chip-based genotyping was higher than the error rate for LDC genotypes.

The non-missing genotypes between each pair of call sets had less than 1% heterozygote discordance (Table 2c). IBC and PBC call sets had low discordance, with *h*_*e*_ = 0.42%. The PBC and LDC call sets also had similar discordance, with *h*_*e*_ = 0.35% and 0.41% respectively. The two LDC call sets were the least discordant, with *h*_*e*_ = 0.30%. IBC and LDC call sets had higher heterozygous discordance, with *h*_*e*_ = 0.93% between IBC and LDC without flanking haplotypes, and *h*_*e*_ = 1.01% between IBC and LDC with flanking haplotypes. These mismatch rates were consistent with the above comparisons with GWAS genotypes and between sequence replicates (Table 2).

Complex calling algorithms called additional genotypes at sites that had missing calls at less complex calling algorithms (Table 3a). To evaluate specifically the quality of these additional sites, we calculated the heterozygous mismatch rates with GWAS genotypes (Table 3b). Comparing each algorithm with progressively more complex alternatives at the 378 on-target variant sites with GWAS information, we observe that the PBC call set contained 15,727 (5.68%) more heterozygous genotypes than the IBC call set, with *h*_*e*_ = 0.85%. Thus PBC generates high-quality genotypes at most sites that cannot be called with IBC. LDC without flanking haplotypes generated 3,113 (1.06%) while LDC with flanking markers generated 3,664 (1.25%) more heterozygous genotypes than PBC. Mismatch rates in these extra genotypes varied widely between the two settings; calls from LDC without flanking markers had a mismatch rate of 2.41% while calls from LDC with flanking markers had an error rate of 0.71% (Table 3b).

### Alternative implementations of variant callers

To evaluate the consistency of these observations across other implementations of variant callers, we analyzed the same dataset using GATK UnifiedGenotyper. We generated individual-based (G-IBC) and population-based (G-PBC) call sets. The G-IBC call set contained 34,704 variants with Ts/Tv 2.21, while the G-PBC call set contained 33,696 variants with Ts/Tv 2.23. Each call set contained about 32% singletons: the G-IBC call set contained 11,001 singletons with Ts/Tv 2.13, and the G-PBC call set contained 10,678 singletons with Ts/Tv 1.77. The proportion of singletons was substantially higher in our IBC call set (59.36%) generated using glfSingle and PBC call set (57.73%) generated using glfMultiples, as well as in previous analyses of the same dataset (60.32%) [26] using the SOAP caller [15]. Since a high proportion of singletons identified by glfSingle and glfMultiples have been experimentally replicated or validated (see above), GATK UnifiedGenotyper is conservative when calling singletons. Nevertheless, G-IBC identified about 3% more singletons than G-PBC and these had significantly higher Ts/Tv, replicating the pattern observed in our analyses using glfSingle and glfMultiples.

### Multi-allelic variants

IBC identified 523 on-target SNPs with more than one non-reference allele. Of these, 513 SNPs (1.87% of 27,500 IBC SNPs) had two non-reference alleles (triallelic) and 10 had three non-reference alleles. Following the population genetics calculations used in Nelson *et al*. [26], we predicted that ~0.9% of variants would be triallelic and that a third allele would be called at 0.5% of biallelic sites due to sequencing error. Under a model of homogeneous mutation rate, we would thus expect a proportion of ~1.4% observed triallelic SNPs. Similar to others [26,35], we observed an excess of triallelic SNPs.

Most of the triallelic variants were rare: 205 (38.53%) had two singleton non-reference alleles, and 253 (47.56%) had one singleton non-reference allele and one more common non-reference allele. For the 10 SNPs showing all four alleles, 8 had at least one singleton non-reference allele. Nelson *et al.* [26] validated 10 out of 10 singleton triallelic variants from the same dataset. Among the 523 multi-allelic variants called by IBC, PBC called 509 biallelic, identifying the non-reference allele with more information (higher allele frequency or higher read depth). PBC identified the remaining 14 multi-allelic SNPs as monomorphic.

### Computational burden

The computational burden of variant calling increases when the algorithm aggregates more information across individuals and sites. Hence IBC is the fastest algorithm and LDC is the slowest. IBC used about 250 CPU-hours to generate all variants for all 7,842 individuals, while PBC used 400 CPU-hours. For IBC, each individual at a specific genomic region can be analyzed in parallel. For PBC, all individuals have to be considered jointly, but genomic positions are independent and can be analyzed in parallel. In terms of memory usage, IBC consumed negligible memory since it only needed to read in the genotype likelihoods for one position per individual. For PBC, memory consumption increased roughly linearly with sample size. To analyze our dataset with 7,842 individuals, the maximum memory usage was 7.9 Gb. In our down-sampling analyzes, sample sizes of 1,000 and 5,000 consumed 1.1 Gb and 5.3 Gb memory respectively.

The LDC model considers all haplotypes jointly, with run time increasing in quadratic scale with the number of haplotypes included in the reference panel, which is the state space of the underlying Hidden Markov Model. Other factors affecting run time included length of each haplotype, number of iterations, and total sample size. We performed LD-aware calling per gene for 15,684 haplotypes at 202 genes, using a reference panel size of 200 for 30 iterations. After running PBC, LDC with flanking haplotypes took about 3000 CPU-hours. Without flanking haplotypes, LDC took about 2000 CPU-hours. To speed up the process while retaining sufficient LD information, LDC can be run in parallel on larger genomic regions, such as a 1Mb region or a chromosome. Memory usage increased linearly with the number of variants in the gene: each gene contained 300 to 2,000 variant sites after adding GWAS flanking genotypes, with the memory required for running LD-aware algorithm ranging from 45 Mb to 300 Mb.

We performed all analyses on a Dell C6100 blade server with four discrete dual 6-core Intel Xeon X5660 CPUs at 2.80 GHz. 128 GB RAM and 1 TB of local SATA disk were available on this system.

## Discussion

We performed an extensive comparison between calling algorithms of various complexity on a large sequencing dataset capturing exons of 202 drug-targeted genes with mean coverage of 24x. As a result of the capturing process necessary for targeted sequencing, we observed a wide range of coverage per targeted position, echoing the outcomes of other exome sequencing studies aiming at high coverage [10,36]. Thus, our work provides general guidelines for using variant calling algorithms on exome and targeted sequencing datasets.

Existing calling algorithms aggregate different levels of information from sequence reads. We considered three major groups of likelihood-based models: (1) Individual-based single marker caller (IBC) uses aligned reads at each marker per individual, (2) population-based single marker caller (PBC) uses aligned reads at each marker for all samples to estimate population allele frequency, (3) LD-aware genotype refinement caller (LDC) uses linkage disequilibrium information from loci surrounding each called variant. Many different approaches exist for each model; each uses a variation of individual-based, population-based or haplotype-based priors. Previous studies have shown comparable performance between glfSingle/glfMultiples and earlier versions of the GATK UnifiedGenotyper [37]. By comparing sets of IBC and PBC from the same developer, we observed excess high-quality singletons in individual- over population-based algorithms.

Comparing filtered call sets of identical size (27,500) for each caller, IBC discovered more rare variants than PBC. In particular, at lower coverage, IBC was able to identify more high-quality singletons than PBC. We replicated this result twice, in a second dataset with higher coverage and in the original dataset using a different approach of the callers. We observed that the ability of PBC to detect singletons depended on sample size: With increasing sample size, PBC identified fewer singletons, and the quality of the identified singletons decreased. This advantage of IBC over PBC can be partly explained by the fact that in larger samples, singletons have an allele frequency < 0.001. Hence the prior for a site being a singleton is stronger in the individual-based caller and less evidence is required to call a singleton.

While we found significant differences between caller-specific sites, IBC and PBC call sets had >99% concordance at the high-quality, non-missing heterozygous genotypes. Our validation experiment confirmed all selected IBC-specific singletons, with very few unconfirmed singletons in the PBC call set. Moreover, most PBC-specific singletons were in the IBC unfiltered (complete) call set. We observed the same trend of IBC generating an augmented set of singletons in high coverage sequencing data (>120x), where IBC almost doubled the number of high-quality singletons at sites with local low coverage (<10x).

Furthermore, only IBC was capable of identifying polymorphisms with more than one non-reference allele, which led to discovery of an additional 1.9% of rare alleles in the sample. The excess of triallelic sites over the theoretical prediction of 1.4% is likely the result of heterogeneity of mutation rate due to sequence context and genomic environment. Existing associations between multiallelic variants and disease phenotypes [38,39] suggest that properly accounting for such variants can increase the power of a sequencing study.

While IBC had strengths in identifying singletons, PBC generated better overall genotype quality. At common variants, PBC genotypes overcame low coverage at specific samples, achieving fewer missing genotypes and higher accuracy than IBC calls. The discordance between IBC and GWAS genotypes was low (0.82%), but more than two times higher than the GWAS discordant rates of the other call sets.

LDC achieves even higher genotype accuracy than IBC and PBC by using haplotype information to impute missing genotypes from an existing single-marker call set. Imputation is typically more effective with longer haplotypes. In our study, we created long haplotypes by combining sequencing data with SNPs from previous GWAS genotyping chips. LDC with such flanking haplotypes achieved the highest accuracy and the least missing genotypes. As targeted sequencing studies might not have chip data to generate long haplotypes, we studied if LDC would still improve genotype accuracy with haplotypes based only on the sequencing data. Without flanking haplotypes, LDC had fewer missing data at the common variants over PBC, yet with a slightly higher mismatch rate. In particular, the additional heterozygote genotypes at common GWAS markers had a high mismatch rate of 2.43%, despite an overall mismatch rate of 0.39%. This suggested that using LDC on short haplotypes to impute missing genotypes created a relatively large number of imputation errors. Comparison between sequence replicates further demonstrated that LDC without flanking haplotypes had minimal benefit over PBC. As LDC imposes a considerable computational burden, it seems questionable whether this caller should be used when flanking haplotypes are not available.

## Conclusions

In summary, while IBC generated high quality unique singletons, as well as multiallelic variants, its resulting call set contained more missing genotypes and genotyping errors at common variants. PBC calls showed a substantial decrease in the number of missing genotypes and errors over IBC calls at these variants. Only when flanking haplotypes were available, LDC calls showed noticeable refinement of PBC genotypes, resulting in a call set with the highest concordance with GWAS genotypes and between experimental replicates. Therefore, IBC had strengths in calling extremely rare variants, while PBC combined with LDC had strengths in calling the more common variants.

Based on these results, we recommend a two-fold calling strategy for targeted sequencing studies with medium to high coverage in a large sample. We recommend first to use a population-based single marker caller to generate accurate common variants and most of the rare variants. Second, we recommend using individual-based single marker caller to enrich the call sets with additional singletons. If flanking markers around targeted regions are available, despite the computation burden, we recommend using LD-aware caller to refine and impute population-based calls at high accuracy, resulting in a complete call set.

### Availability of supporting materials

The documentation, dataset, figures and tables supporting the results of this article are included in Additional files 1 and 2: Dataset S1.

## Additional files

Additional file 1:
**Contains additional text,** **Figures S1 to S3** **and** **Tables S1 to S2** **that supplement the description of analyses in the main text.**

Additional file 2:
**Contains the list of 1,035 variants not previously found in the Nelson** ***et al*** **. analyses of the same dataset [** 26 **].**
