# Supplementary material for: Comparing variant calling algorithms for target-exon sequencing in a large sample
Source: BMC Bioinformatics. 2015 Mar 7;16:75. doi: 10.1186/s12859-015-0489-0 (PMC4359451; doi:10.1186/s12859-015-0489-0)
Supplement: Additional file 1: — Contains additional text, Figures S1 to S3 and Tables S1 to S2 that supplement the description of analyses in the main text. [file 12859_2015_489_MOESM1_ESM.pdf]

Additional File 1 for

Comparing variant calling algorithms for target-exon sequencing data in a large sample

Yancy Lo [1,*], Hyun M. Kang [1], Matthew R. Nelson [2], Mohammad I. Othman [3], Stephanie L. Chissoe [2], Margaret G. Ehm [2], Gonçalo R. Abecasis [1], Sebastian Zöllner [1,4]

[1]Department of Biostatistics, University of Michigan, Ann Arbor, MI
[2]GlaxoSmithKline, Quantitative Sciences, RTP, NC
[3]Department of Ophthalmology, University of Michigan, Ann Arbor, MI
[4]Department of Psychiatry, University of Michigan, Ann Arbor, MI

This PDF file includes:

Additional Text

Figures S1 to S3

Tables S1 to S2

Other supplementary material for this manuscript includes the following:
Additional File 2: Database S1 as zipped archive

Pre-variant calling data processing

Sequence read data

We aligned reads using BWA 0.5.9 (http://bio-bwa.sourceforge.net) with human genome build 36 as reference. Average mapping rate was 99.7%; 98.5% of reads were properly paired. Using Picard (http://picard.sourceforge.net/) we identified and removed 21% duplicate reads. We recalibrated the base quality scores using GenomeAnalysisTK-1.0.5974 (http://www.broadinstitute.org/gsa/wiki/index.php/Base_quality_score_recalibration).[1]

Genotype data

We combined genotype data from previous GWASs typed on Illumina 300k, 550k, 610k and Affymetrix 500k and 6.0 using PLINK.[2] We identified 378 GWAS variants on the targeted regions. At these variants, we confirmed reference allele for A/T and G/C variants using sequencing calls and respective allele frequencies, and there were no strand flip issues. We discarded a small number of genotypes at the flanking regions based on ambiguous strand information.

Variant quality control

Initial filtering

We applied to each call set initial filters, which were based on read alignments at variant sites and summary statistics of each site. In particular, at each polymorphic site, we computed several *Z*-score test statistics of read alignments, including strand bias, allele balance and alternative allele inflation, with the detailed statistical tests described below. A SNP with extreme *Z*-scores indicate bias from mapping or sequencing artefacts which likely lead to false positive calls. Cutoffs for each filter followed from the ones used in the NHLBI GO Exome Sequencing Project.[3] We further imposed an indel filter, which filtered SNPs located within 5 base pairs of known insertions or deletions from 1000 Genomes low-coverage CEU data (July 2010 release). We detected sites with excess heterozygosity than expected under Hardy-Weinberg equilibrium, calculated using inbreeding coefficient, described below. For LD-aware calls, we imposed an additional R^2 quality control criterion by filtering the sites with estimated squared correlation less than 0.7 between true allele counts and estimated allele counts. Here we describe in detail the filters used:

1. Strand bias: Conditioned on the site being biallelic, strand bias refers to higher than expected frequency of observing the alternate allele on the forward or the reverse strand. Specifically, the strand bias filter counts the number of reference and alternate alleles on each strand as a 2-by-2 contingency table. Under the null hypothesis, a genuine polymorphism should have the alternate allele observed equally often from forward and reverse strands. Therefore, the strand bias filter discards sites with normalized $|Z|$-score greater than 10 or absolute correlation greater than 0.15, which suggest strong association between strand and the allele observed.

2. Allele balance: Allele balance measures the ratio between allele counts from genotype calls and estimated allele counts calculated from individual sequence depth and likelihoods (http://genome.sph.umich.edu/wiki/Genotype_Likelihood_Based_Allele_Balance). A small ratio indicates bias towards certain alleles at a called polymorphic site, which is likely to be false positives. We imposed a lower bound of 67% on the allele balance ratio for good quality SNPs.

3. Alternate allele inflation: Alternate allele inflation is a composite measure of base quality inflation and alternate allele quality inflation. We count the number of third and fourth alleles observed at a biallelic site and test it against the expected value where third and

fourth alleles only occur due to sequencing error. Large normalized $|Z|$-score of this test indicates there are more non-reference, non-alternate bases than expected by base quality, suggesting that base quality is over-recalibrated due to alignment artefacts. Alternate allele quality inflation measures the normalized deviation of the number of alternate allele calls from the actual number of alternate bases observed from the reads. A small $|Z|$-score provides stronger support of the site being polymorphic, as the alternate base is observed much more frequently than the other two bases besides the reference. The composite alternate allele inflation statistic is the sum of the two $|Z|$-scores described above. We filtered out sites having absolute composite score greater than 5, which means they are called polymorphic because of alignment artefacts that lead to inflated quality scores.

4. Excess heterozygosity: We measured deviation from Hardy-Weinberg equilibrium (HWE), in particular the excess of heterozygotes, by calculating the inbreeding coefficient F for each marker, where

$$F = 1 - \frac{Observed\ number\ of\ heterozygous\ genotypes}{Expected\ number\ of\ heterozygous\ genotypes}$$

The expected number of heterozygotes comes from assuming HWE, such that

$$E(het) = 2p(1-p)N$$

Here N denotes the sample size. F ranges from $(-\infty, 1]$, with positive values representing markers with fewer heterozygotes than expected. $F = 0$ means the marker is in perfect HWE. Negative F denotes an excess heterozygotes at that marker. We set the cut-off at -0.1, meaning that we discard markers with more than 10% heterozygotes observed than expected under HWE.

Support Vector Machine (SVM) filtering

Second, based on the initial set of variant quality metrics, we used a support vector machine (SVM) approach to generate a summary quality score for each variant site.[4] This approach was also applied in filtering and generating consensus calls in ESP and 1000 Genomes Project.[3, 5] The SVM identifies a hyperplane separating a training set of good calls and bad calls and scores each variant site to reflect the distance of the SNP from this hyperplane. Good calls and bad calls are classified by contrasting the initial quality statistics between the SNP calls and the SNPs in positive and negative training sets respectively. We used HAPMAP3 and OMNI variant sites as positive training sets, and the calls that did not pass more than two initial quality metrics as the negative training set.

Genotype filtering

Third, after selecting a fixed call rate of 27,500 top-ranked variants per call set from SVM classification, we applied filters to individual genotypes to ensure quality of all genotypes under comparison, given each top-ranked variant site. From genotypes called by individual-based single marker caller (IBC), we removed and marked as missing the genotypes with PHRED quality score less than 20; we also removed genotypes with genotype depth less than 7x. The quality of genotypes called by population-based single marker caller (PBC) is less affected by genotype depth, hence we only filtered based on PHRED genotype quality < 20. Analogously, we filtered LD-aware genotype calls with a posterior probability ratio < 99:1 between the genotype with the highest posterior probability and the genotype with the second highest posterior probability.

Validation experiment

We performed an independent Sanger capillary sequencing to validate singleton variants identified by IBC and PBC. We considered the singletons carried by individuals from the CoLaus study.[6] Within this subset of individuals, we sampled from the top-ranked 27,500 variants 32 singletons called by only IBC and 41 singletons called by only PBC. We further extended the experiment to sequence some caller-specific singletons beyond our defined SVM ranking cutoff. For variants ranked between 27,501 and 29,000 in each call set, we sampled 16 IBC-specific singletons and 12 PBC-specific singletons from the CoLaus individuals. Since IBC called more variants than PBC, we sampled an additional 23 IBC-specific singletons at the tail of the SVM rankings ($> 29{,}000$). We performed capillary sequencing on these 124 singletons on the individuals carrying the heterozygous genotype.

After PCR amplification of sequences of the 124 singletons using designed primers, we performed Sanger sequencing on the PCR products. We performed both steps at the University of Michigan facilities. We designed PCR primers using NCBI Primer-BLAST program. In case the program was not able to pick the primers, we manually designed primers sequences and ran them through BLAST search for specificity. We amplified PCR amplicons using OneTaq hot start 2X mixes (NEB, USA) with standard or GC buffer depending on the GC contents of the sequences. For samples that did not amplify in the first round, we assigned them new primers before repeating amplification. We set up PCRs using GeneAmp PCR System 9700 (Applied Biosystems, USA). We ran aliquots of the amplicons on 1% TBE agarose gel with Sybr Safe DNA Gel Stain and viewed them in UVP or Typhoon 9000 to visualize the amplicons and to check the quality and the quantity of the amplified bands. We ran other PCR amplicons on the Agilent Bioanalyzer 2200 TapeStation (Agilent, USA) using the D1K screen tapes. We diluted amplicons before performing Sanger sequencing with the selected primers. We verified sequencing chromatogram data using Sequencher 5.1 demo (CGC, USA). We reported alleles by inspecting peaks on each chromatogram.

Among the 124 reactions performed, 3 failed. Among the 121 successful reactions, 71 were expected heterozygotes that passed our SVM quality control threshold. In this category, 3 out of 41 (7.32%) PBC-specific singletons were found to be homozygous reference, while all 30 IBC-specific singletons were confirmed. This difference in error rates between IBC and PBC was not statistically significant (Fisher's exact p-value = 0.258). Beyond our defined quality control threshold, at variants ranked between 27,501 and 29,000 in each call set, 4 out of 16 IBC-specific and 1 out of 12 PBC-specific singletons were not confirmed. At the tail of the SVM-ranked IBC call set, 4 out of 22 IBC-specific singletons were found to be homozygous reference, corresponding to a calling accuracy of about 82% for IBC at the sites of lowest quality (Table S2).

Evaluation of singletons on additional data set

We applied individual-based variant calling (IBC) on 3,142 individuals from the AMD Consortium targeted sequencing dataset.[7] This sample was sequenced at 57 genes at 10 AMD loci, at 127.5x. Despite high average coverage, we observed highly heterogeneous coverage across targeted genes (Figure S2). Several genes are covered at less than or close to 10x. The population-based variant calling (PBC) of the same sample were previously performed and published by Zhan *et al.*[7] After filtering the IBC call set using the same initial filters as in the PBC analyses, we compared the singleton calls identified by IBC and PBC.

Across the dataset, IBC called 1,913 additional singletons with genotype quality >10 compared to PBC. These additional singletons had a Ts/Tv ratio of 1.63. Interestingly, the additional singletons with high quality were located in regions with low coverage. We found that at coverage < 10, and with an additional genotype quality filter of > 10, IBC identified

864 additional singletons not found in the PBC call set, with Ts/Tv = 2.18 (Figure S3 top). At the same genotype depth and quality thresholds, IBC and PBC shared 911 singleton variant calls with Ts/Tv = 2.13 (Figure S3 bottom). When we relaxed the genotype depth threshold to < 20x, IBC identified 1,360 additional singletons with Ts/Tv = 1.90. At the same thresholds, IBC and PBC shared 2,745 singletons with Ts/Tv = 2.07.

References

1 DePristo MAea: A framework for variation discovery and genotyping using next-generation DNA sequencing data. 2011; **43:** 491-498.

2 Purcell S, Neale B, Todd-Brown K *et al:* PLINK: a tool set for whole-genome association and population-based linkage analyses. *The American Journal of Human Genetics* 2007; **81:** 559-575.

3 Tennessen JA, Bigham AW, O'Connor TD *et al:* Evolution and Functional Impact of Rare Coding Variation from Deep Sequencing of Human Exomes. 2012; **337:** 64-69.

4 Jun G, Wing MK, Abecasis G, Kang HM: An efficient and scalable analysis framework for variant extraction and refinement from population scale DNA sequence data. *In preparation* .

5 The 1000 Genomes Project Consortium: An integrated map of genetic variation from 1,092 human genomes. *Nature* 2012; **491:** 56-65.

6 Nelson M, Ehm M, Wegmann D *et al:* An abundance of rare functional variants in 202 drug target genes sequenced in 14,002 people. 2012; **337:** 100-104.

7 Zhan X, Larson DE, Wang C *et al:* Identification of a rare coding variant in complement 3 associated with age-related macular degeneration. *Nat Genet* 2013; **45**:1375–1379.

Supplementary Figures

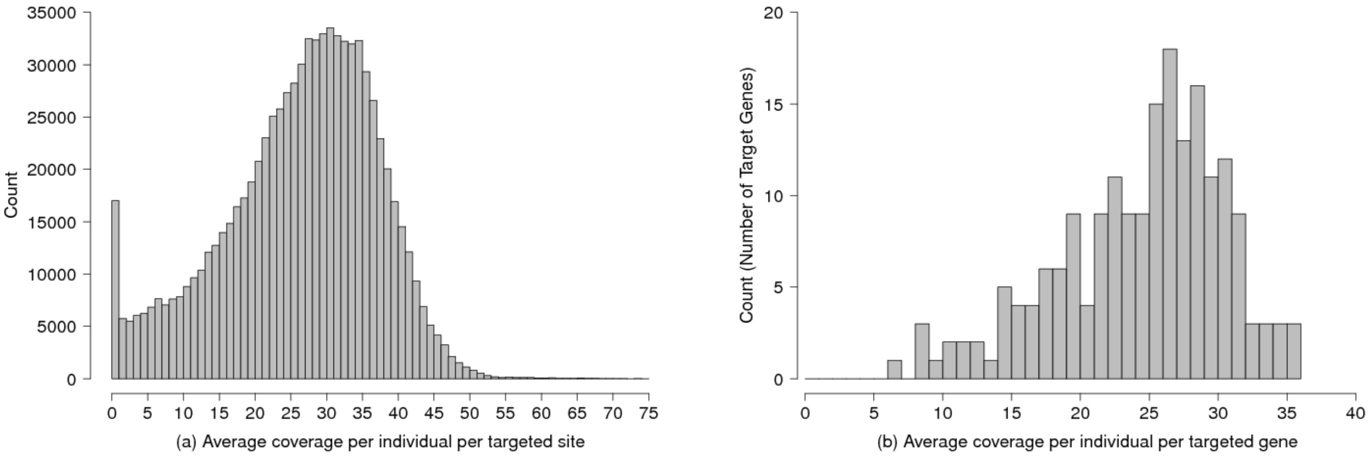

Figure S1: Distribution of average coverage of sequence read data from 7,842 unrelated European individuals. The next-generation sequencing data was part of a large-scale targeted sequencing experiment generated for the purpose of identifying variants associated with 12 common diseases and cardiovascular and metabolic phenotypes, previously described in Nelson *et al*.[6] This experiment targeted 2,218 exons of 202 genes of potential drug interest, covering 864kb (1%) of the coding genome (a) per individual per targeted genomic position, (b) per individual per targeted gene. The overall mean coverage is 24x.

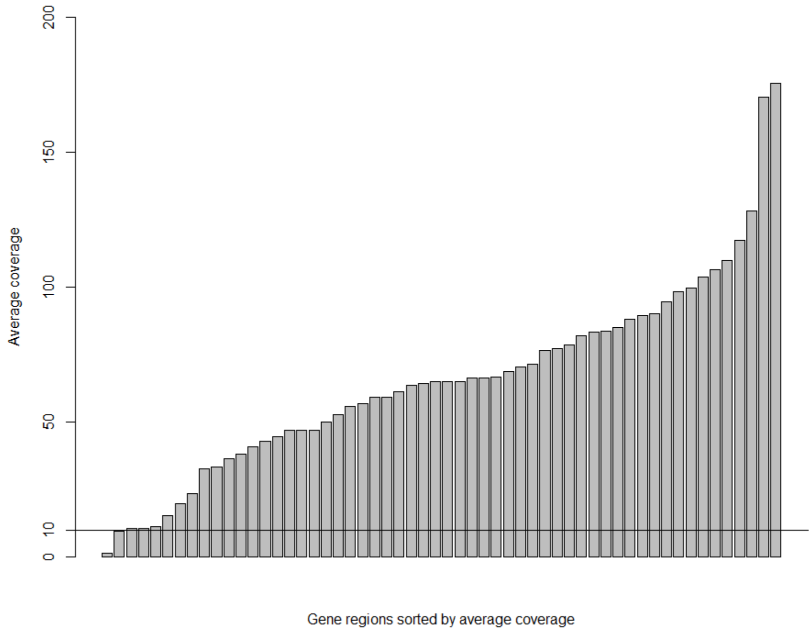

Figure S2: Average coverage at the 57 targeted genes in the AMD sequencing study[8], ranked by average coverage per individual per gene position. Overall average coverage across the whole targeted region was 127.5x. Horizontal line denote 10x coverage.

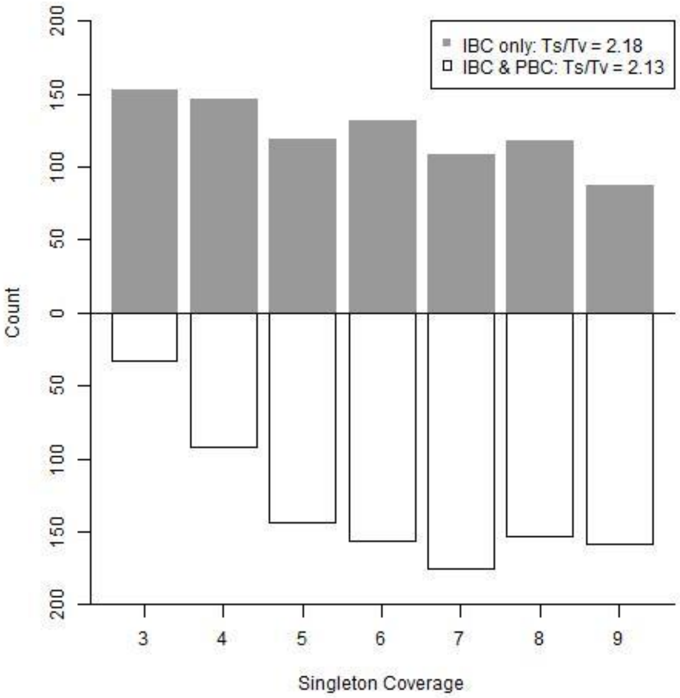

Figure S3: Distribution of AMD[7] singleton site coverage at the singleton-carrier at coverage < 10. All singletons shown in the figure have genotype quality > 10. Top (gray): singletons identified by IBC only, Ts/Tv = 2.18. Bottom (white): singletons identified by both IBC and PBC, Ts/Tv=2.13.

a

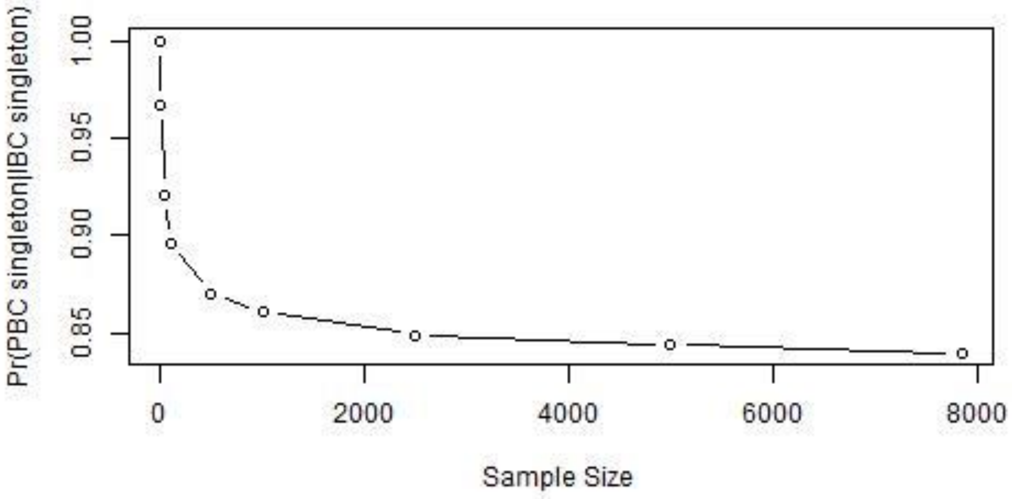

b

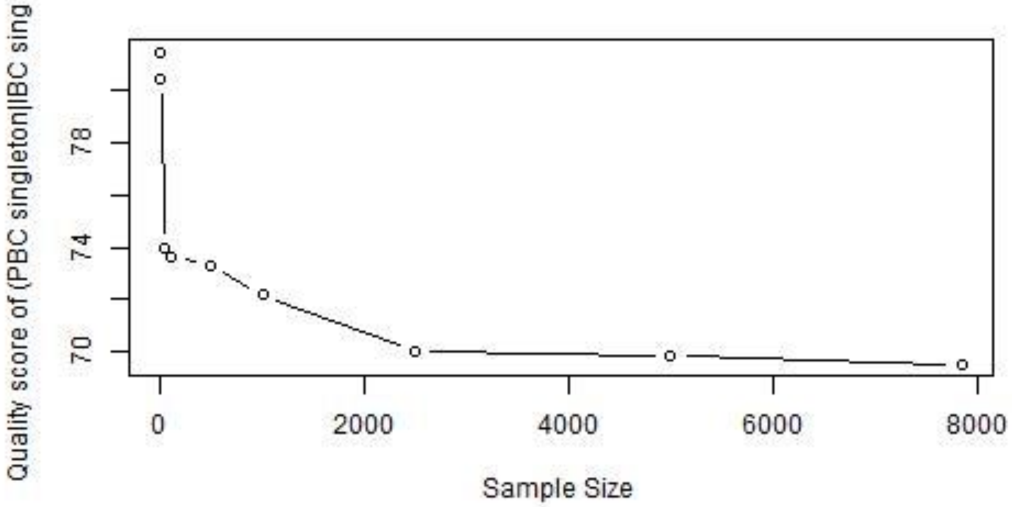

Figure S4: (a) Proportion of IBC singletons identified by PBC at different sample sizes, **(b)** Average quality score of IBC singletons identified by PBC at different sample sizes.

Supplementary Tables

Table S1: Quality of call sets assessed by transition-to-transversion ratio (Ts/Tv), broken down by variant class and frequency. Ts/Tv of each set at the top-ranked 27,500 SNPs via SVM classification were higher than the respective unfiltered call sets. Under a uniform Ts/Tv prior for all algorithms, IBC call set attained a higher Ts/Tv than the other call sets. Ts/Tv was higher at exonic variants and at known variants than at intronic variants and novel variants. Variants were classified using the ANNOVAR nomenclature (http://www.openbioinformatics.org/annovar/annovar_gene.html), with "Splice" including the splicing only sites, while the splice sites that lead to a stop codon were in the "Stop" class. *"Flank" refers to the upstream/ downstream variants within 50bp of the transcription site, as designed in the capture experiment described in Nelson *et al.*[6]

Caller	Class	All SNPs					Singletons	
		#SNPs	% dbSNP	Known Ts/Tv	Novel Ts/Tv	Overall Ts/Tv	#SNPs	Ts/Tv
IBC	**Total**	**27500**	**25.72%**	**3.02**	**2.54**	**2.71**	**16325**	**2.57**
	Nonsynonymous	6522	26.37%	2.92	2.30	2.50	4264	2.34
	Synonymous	4363	37.06%	14.90	5.31	5.60	2461	5.34
	Splice	130	16.15%	1.10	1.53	1.45	86	1.46
	Stop	163	20.25%	1.54	1.89	1.88	126	1.74
	UTR	10261	22.06%	2.50	2.30	2.39	5915	2.24
	Intronic	5610	23.46%	2.58	2.41	2.50	3213	2.49
	Flank*	341	24.93%	2.04	2.05	2.08	189	2.10
	Intergenic	110	14.55%	1.29	2.03	2.00	71	1.73
PBC	**Total**	**27500**	**26.87%**	**3.02**	**2.45**	**2.59**	**15877**	**2.44**
	Nonsynonymous	6547	27.19%	2.81	2.24	2.38	4222	2.25
	Synonymous	4377	38.15%	14.80	5.11	5.33	2415	5.16
	Splice	117	17.95%	1.33	1.74	1.66	76	1.81
	Stop	157	21.02%	2.30	1.88	1.96	119	1.77
	UTR	10285	23.11%	2.47	2.22	2.28	5759	2.12
	Intronic	5558	24.88%	2.65	2.29	2.37	3057	2.30
	Flank*	349	31.52%	2.33	1.91	2.03	160	1.86
	Intergenic	110	14.55%	1.67	2.03	1.97	69	1.76
LDC	**Total**	**27500**	**26.85%**	**3.01**	**2.45**	**2.59**	**15857**	**2.44**
	Nonsynonymous	6574	27.17%	2.78	2.24	2.37	4235	2.24
	Synonymous	4375	38.08%	15.05	5.13	5.37	2419	5.19
	Splice	119	17.65%	1.33	1.65	1.59	77	1.75
	Stop	157	21.02%	2.30	1.88	1.96	119	1.77
	UTR	10273	23.10%	2.46	2.22	2.28	5741	2.13
	Intronic	5549	24.82%	2.60	2.30	2.37	3044	2.31
	Flank*	342	32.75%	2.29	1.91	2.03	152	1.81
	Intergenic	111	14.41%	1.67	1.97	1.92	70	1.69
LDC+F	**Total**	**27500**	**26.81%**	**3.00**	**2.45**	**2.58**	**15869**	**2.44**
	Nonsynonymous	6570	27.12%	2.78	2.25	2.38	4235	2.25
	Synonymous	4378	38.05%	15.00	5.14	5.36	2419	5.20
	Splice	120	17.50%	1.33	1.61	1.55	78	1.69
	Stop	157	21.02%	2.30	1.88	1.96	119	1.77
	UTR	10265	23.08%	2.47	2.21	2.27	5742	2.11
	Intronic	5558	24.79%	2.60	2.29	2.36	3053	2.29
	Flank*	341	31.67%	2.38	1.88	2.02	153	1.73
	Intergenic	111	14.41%	1.67	1.97	1.92	70	1.69

Table S2: Validation experiment results. Independent Sanger capillary sequencing experiment showed 0 out of 30 errors from IBC-specific singletons and 3 out of 41 (7.32%) errors from PBC-specific singletons, sampled from the respective caller-specific singleton variant calls that passed quality control. The difference between IBC and PBC error rates is not statistically significant (Fisher's exact p-value = 0.258). Sampling from the low quality caller-specific singletons, Sanger sequencing reported 4 out of 16 IBC-specific and 1 out of 12 PBC-specific singleton errors. At the tail of the SVM-ranked IBC call set, 4 out of 22 IBC-specific singletons were found to be homozygous reference, corresponding to a calling accuracy of about 82% for IBC at the sites of lowest quality.

SVM ranking	Caller	Total reactions	Failed reactions	Confirmed	Not confirmed
≤ 27,500	IBC-specific	32	2	30	0
	PBC-specific	41	0	38	3
27,501-29,000	IBC-specific	16	0	12	4
	PBC-specific	12	0	11	1
>29,000	IBC-specific	23	1	18	4
